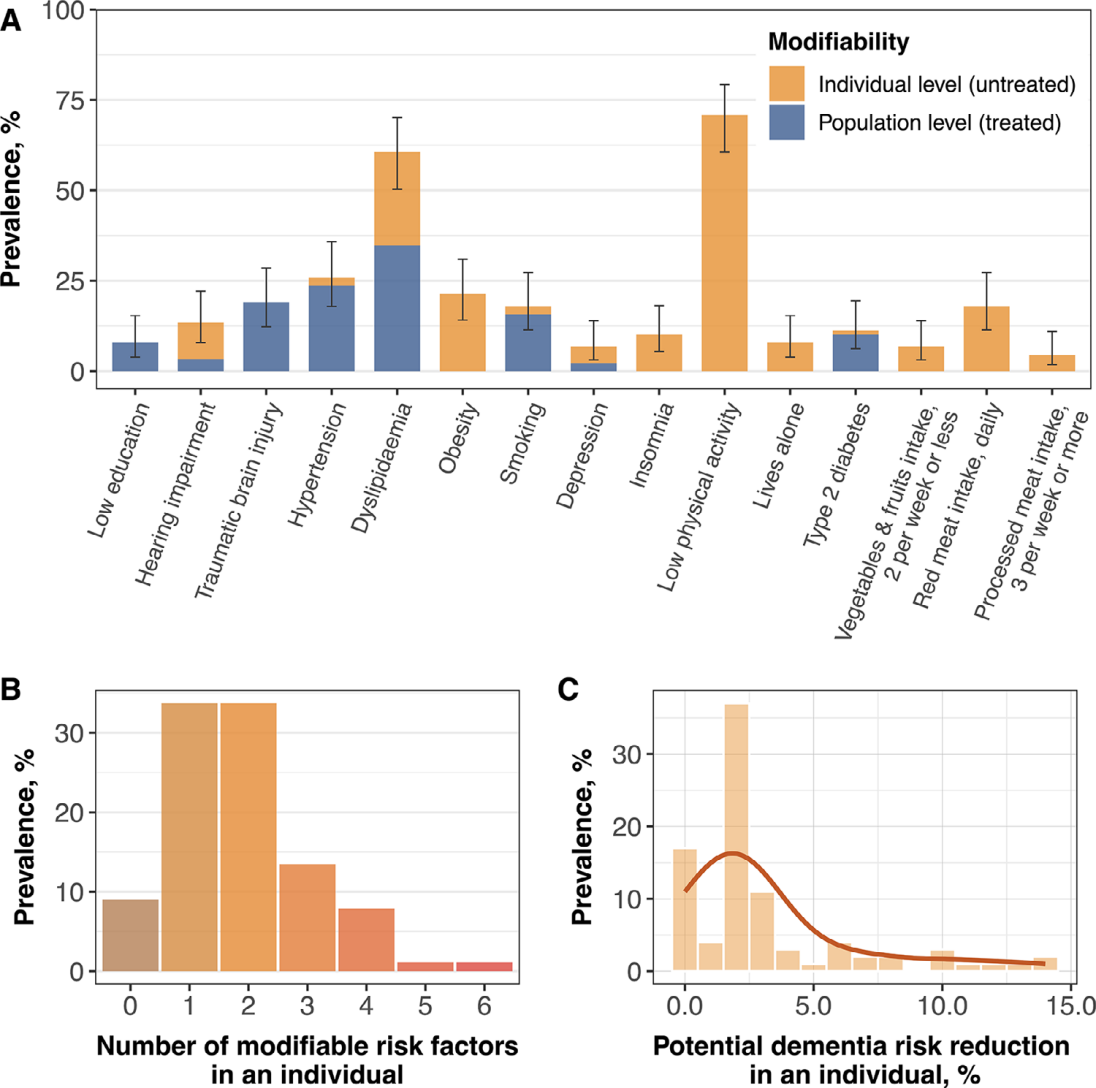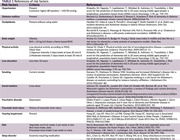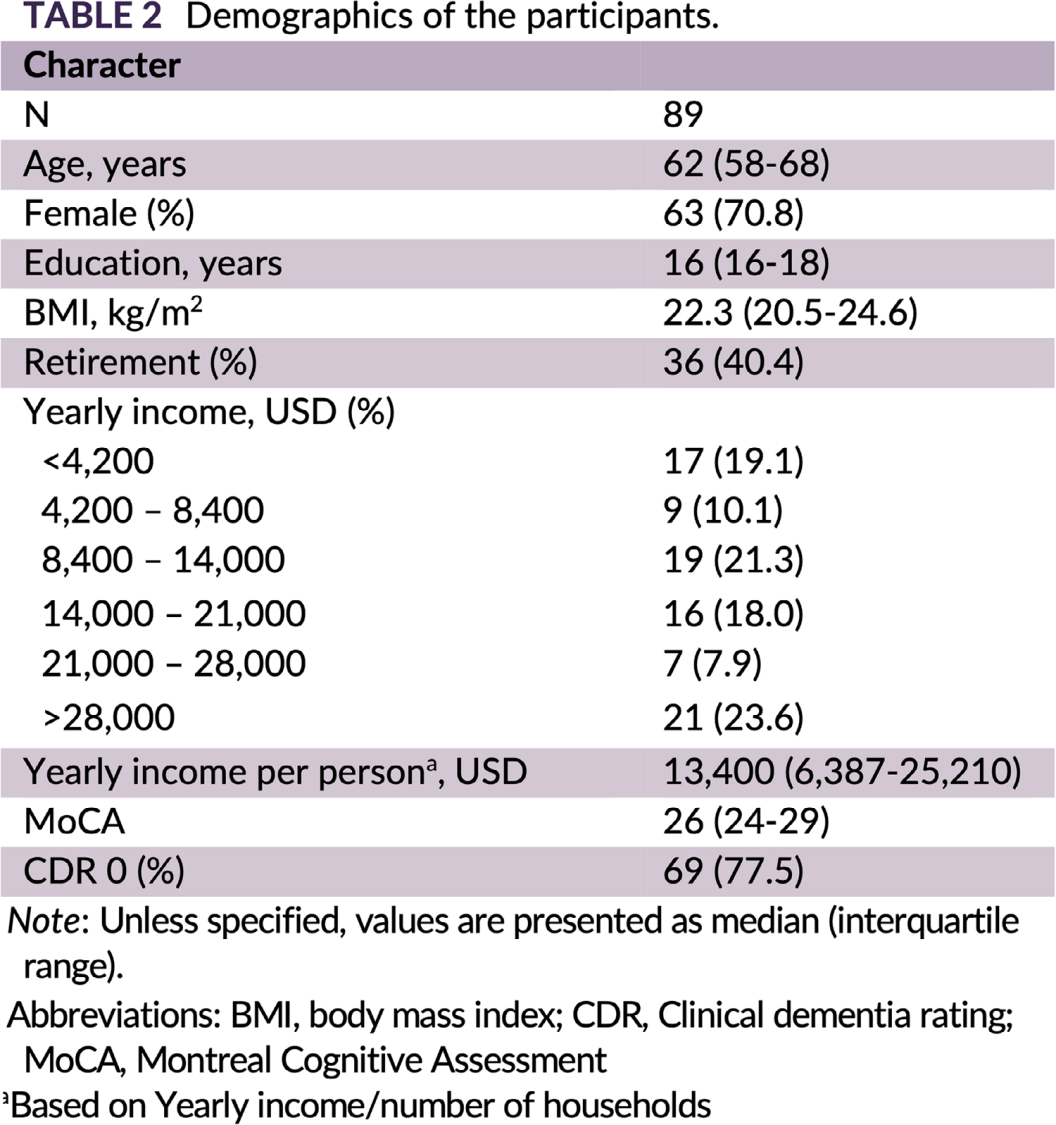# Prevalence of modifiable risk factors in cognitive aging cohort participants in Bangkok, Thailand

**DOI:** 10.1002/alz.092176

**Published:** 2025-01-09

**Authors:** Pannita Sengpanich, Poosanu Thanapornsangsuth, Thiravat Hemachudha

**Affiliations:** ^1^ Division of Neurology, Department of Medicine, Faculty of Medicine, Chulalongkorn University, Bangkok Thailand; ^2^ Thai Red Cross Emerging Infectious Diseases Health Science Centre, King Chulalongkorn Memorial Hospital, Bangkok Thailand; ^3^ Memory Clinic, King Chulalongkorn Memorial Hospital, Bangkok Thailand; ^4^ Chula Neuroscience Center, King Chulalongkorn Memorial Hospital, Bangkok Thailand; ^5^ Memory Clinic, King Chulalongkorn Memorial Hospital, The Thai Red Cross Society, Bangkok Thailand

## Abstract

**Background:**

Dementia poses an escalating socioeconomic challenge, yet evidence suggests potential prevention through proactive correction of risk factors and multidomain interventions. In cost‐conscious Thailand, targeting prevalent risk factors may offer the most feasible strategy for disease prevention, ultimately alleviating healthcare burdens.

**Method:**

Non‐demented participants were consecutively selected from the INDIE cohort, a longitudinal study on cognitive aging at Chulalongkorn University in Bangkok, Thailand. A 40‐minute interview encompassed symptoms, risk factors, social determinants of health, and quality of life. Modifiable risk factors for dementia were identified through an comprehensive literature review (Table 1) and categorized as modifiable at the individual level (e.g., still uncontrolled in the participant) or at the population level (indicating sufficient efforts by the participant). Beyond examining prevalence in the entire cohort, we explored the number of risk factors present in each participant and assessed the impact of correcting these factors on dementia prevention using figures from the Lancet Commission (Livingston, 2020).

**Result:**

Non‐demented participants were consecutively selected from the INDIE cohort, a longitudinal study on cognitive aging at Chulalongkorn University in Bangkok, Thailand. A 40‐minute interview encompassed symptoms, risk factors, social determinants of health, and quality of life. Modifiable risk factors for dementia were identified through an comprehensive literature review (Table 1) and categorized as modifiable at the individual level (e.g., still uncontrolled in the participant) or at the population level (indicating sufficient efforts by the participant). Beyond examining prevalence in the entire cohort, we explored the number of risk factors present in each participant and assessed the impact of correcting these factors on dementia prevention using figures from the Lancet Commission (Livingston, 2020).

**Conclusion:**

This underscores the necessity for healthcare policies targeting dementia prevention, emphasizing modifiable risk factors like low physical activity. While the impact of correcting these risk factors on an individual may seem modest, it can be substantial for those at high dementia risk, particularly when identified through biomarkers. Limitations include a small sample size and non‐generalizable participant selection; future research should extend to rural communities and impoverished urban areas with larger anddiverse samples.